# Testing a Web-Based Interactive Comic Tool to Decrease Obesity Risk Among Minority Preadolescents: Protocol for a Pilot Randomized Control Trial

**DOI:** 10.2196/10682

**Published:** 2018-11-09

**Authors:** May May Leung, Katrina F Mateo, Sandra Verdaguer, Katarzyna Wyka

**Affiliations:** 1 School of Urban Public Health Hunter College New York, NY United States; 2 Graduate School of Public Health and Health Policy City University of New York New York, NY United States

**Keywords:** mHealth, pediatric obesity, vulnerable populations, minority, diet, child, parents

## Abstract

**Background:**

Childhood obesity is a public health crisis, particularly in low-income, minority populations in the United States. Innovative and technology-enhanced interventions may be an engaging approach to reach at-risk youth and their parents to improve dietary behaviors and feeding practices. However, such tools are limited, especially ones that are theory-based; co-developed with user-centered approaches; tailored to low-income, minority preadolescents; and include parent-focused content.

**Objective:**

The objectives of this study include assessing the feasibility and acceptability and exploring the potential impact of the *Intervention INC* (Interactive Nutrition Comics for urban, minority preadolescents) Web-based tool, which is focused on decreasing childhood obesity risk in black/African American and Latino children aged 9 to 12 years.

**Methods:**

*Intervention INC* is underpinned by the narrative transportation theory, social cognitive theory, and health belief model, and it was co-developed by children and parents from the intended population. The child component consists of a 6-chapter interactive nutrition comic optimized for use on tablet devices, a goal-setting and self-assessment feature, and weekly text/email messages and reminders. The parental component consists of 6 Web-based newsletters, access to the child comic, and weekly text/email messages and reminders. The tool was evaluated using a pilot, single-blind, 2-group randomized controlled study design. Child-parent dyads were randomized to either the experimental or comparison group and assigned to a targeted behavior (increase fruit/vegetable or water intake) based on initial screening questions. Data were collected at 4 time points: baseline (T1), intervention midpoint (T2), intervention endpoint (T3), and 3 months postintervention (T4). Primary measures comprise usage, usability, and feasibility of the Web-based tool. Secondary measures comprise dietary knowledge, preferences, and intake and anthropometric measures (for child) and feeding practices and home food environment (for parent).

**Results:**

Study enrollment was completed in November 2017. A total of 89 child-parent dyads were randomized to either the experimental (n=44) or comparison (n=45) group. Data analysis is currently being conducted.

**Conclusions:**

This study aims to implement and assess an innovative approach to deliver health messages and resources to at-risk minority preadolescents and their parents. If found to be acceptable, engaging, feasible, and a potential approach to improve dietary behaviors, a full-fledged randomized controlled trial will be conducted to assess its efficacy and potential impact.

**Trial Registration:**

ClinicalTrials.gov NCT03165474; https://clinicaltrials.gov/ct2/show/NCT03165474 (Archived by WebCite at http://www.webcitation.org/73122IjgP)

**International Registered Report Identifier (IRRID):**

RR1-10.2196/10682

## Introduction

Childhood obesity continues to be a serious clinical and public health issue in the United States. Over the last three decades, the rate of childhood obesity has tripled, with 17.0% of children aged 2 to 19 years now considered obese [1]. Although the US childhood obesity rate has leveled off in recent years, the challenge remains pronounced among certain populations, particularly in low-income, minority groups. Latino and black/African American children have the highest rates at 21.9% and 19.5%, respectively [1]. This epidemic has profound short- and long-term consequences as it not only leads to negative health outcomes such as type 2 diabetes and cardiovascular disease and can compromise a child’s quality of life but also increases the risk of adult morbidity and mortality [1-6].

Effective yet innovative interventions are needed to capture the attention of children living in a multimedia environment. Visual narratives such as comics may engage today’s youth population around health topics and promote positive psychosocial and behavioral outcomes [7-9]. Moreover, narrative-based health communication interventions can be effective with populations that have strong storytelling traditions such as Latino and African American communities, especially when cultural elements are incorporated [10-13]. Furthermore, the pervasiveness of technology and new media use in children, particularly within black and Latino populations, highlights opportunities and new avenues to engage with this priority population [14-16].

Web-based and technology-enhanced interventions, particularly if developed with user-centered approaches and informed by theory [17], also have the potential to increase access, improve convenience, decrease cost, and increase participant engagement with dietary behavior change strategies, especially among culturally diverse and hard-to-reach communities [18-21]. Indeed, some recent studies have indicated that Web-based programs could improve dietary behaviors in school-aged children [22-25]. However, such interventions tailored to minority youth are limited, especially those which have been co-designed by and/or developed for this population [26-30]. Lifestyle interventions developed to be culturally tailored or culturally relevant have the potential to be more readily adopted by at-risk minority populations [31-34]. Furthermore, this gap in tailored health promotion tools is particularly apparent within the preadolescent population, which is an understudied, yet critical stage of development. Not only does obesity prevalence increase when children transition into adolescence [35] but food preferences and behaviors established during this developmental period often continue into adulthood [36-39].

Despite the increase in childhood obesity studies using technology, mobile health (mHealth), and interactive media, there remain few systematic reviews of this literature and none specific to or tailored for minority/at-risk youth [40-43]. Broad recommendations for future work in this area highlight the need to identify the most effective approaches and strategies to impact behavioral and related health outcomes. In addition, knowledge gaps and challenges related to the implementation and adoption of technology-enhanced interventions exist (eg, identification and recruitment of at-risk, low-income minorities with internet access), which limit their potential effectiveness [40].

The purpose of this paper is to describe the protocol for the implementation and assessment of the *Intervention INC* (Interactive Nutrition Comics for urban, minority preadolescents) Web-based tool, which focuses on decreasing childhood obesity risk in Latino and black/African American children aged 9 to 12 years. This intervention builds on previous research and recommendations for childhood obesity interventions as it is theory-guided, focused on improving key dietary-related health behaviors, delivered via a narrative-based comic medium, and enhanced through an engaging mHealth platform, and it was developed with the intended population (Latino and black/African American preadolescents). The objectives of the study include assessing the feasibility and acceptability and exploring the potential impact of the intervention on dietary behaviors using a pilot, single-blind, 2-group randomized study design.

## Methods

### Study Design

**Figure 1:**
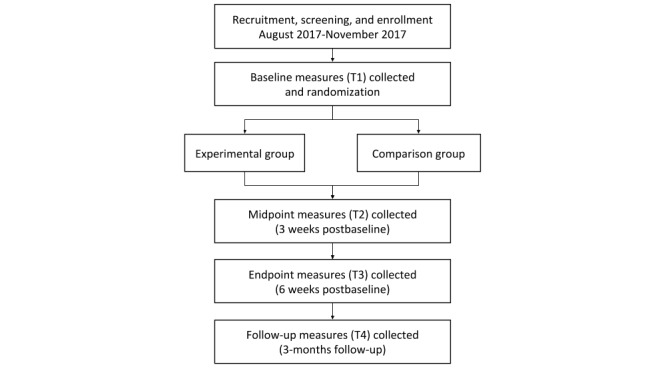
Study design of *Intervention INC*.

The *Intervention INC* study is a pilot, single-blind, 2-group randomized controlled trial (RCT) that evaluated a 6-week intervention, with a 3-month follow-up period (see Figure 1). Child-parent dyads were enrolled into the study on a rolling basis between August and November 2017. Dyads were randomized to either the experimental group, in which the child received a Web-based comic with health messages primarily promoting either fruit/vegetable (F/V) or water consumption or the comparison group, in which the child received Web-based newsletters with health information similarly promoting primarily F/V or water consumption. Parents of both groups received Web-based health newsletters; however, parents in the experimental group were also given access to the child comic. Dyads were blinded to group assignment. Data were collected at 4 different time points: baseline (T1), intervention midpoint or 3 weeks postbaseline (T2), intervention endpoint or 6 weeks postbaseline (T3), and 3-month follow-up postintervention (T4).

The study was approved by the Hunter College institutional review board and is registered with the Clinical Trials Registry (NCT03165474). Adult consent, parental permission, and child assent were obtained at baseline before the commencement of any study procedures.

### Study Population

#### Child

Children residing in New York City (NYC) were recruited based on the following inclusion/exclusion criteria: self-identifies as black/African American and/or Latino; aged between 9 and 12 years (preadolescents) at the time of scheduled baseline visit, reads and speaks in English; has a body mass index (BMI) percentile at or above 5% at baseline (categorized as healthy, overweight, or obese); has regular internet access via a tablet device, mobile phone or computer/laptop; has regular access to a phone with texting capability; is comfortable reading/viewing material on electronic devices; is comfortable speaking with study staff about thoughts/experiences while participating in the study; has no allergies, food aversions, food disorders, or medications with side-effects that may impact participation in the study; does not have a pacemaker or heart condition; and has a legal parent/guardian willing to participate in the study.

It should be noted that original criteria included a BMI percentile at or above 85% (categorized as overweight/obese). Due to recruitment challenges and evidence highlighting that most youth, regardless of BMI status (healthy, overweight, or obese), do not consume the daily recommended amount of fruits/vegetables and water [44-46], the criterion was changed to expand the BMI percentile range to include healthy-weight children (BMI percentile at or above 5%). In addition, the criterion regarding comfort level of child speaking with study staff was added after recruitment began due to observations at initial baseline visits of some children who were unable/unwilling to verbalize their thoughts/experiences.

#### Parent/Guardian

Parents/guardians were recruited based on the following inclusion/exclusion criteria: legal parent/guardian of child willing to participate in the study; reads and speaks in English or Spanish; primarily responsible for preparing/purchasing food for child; has regular internet access via a tablet device, smartphone, or computer/laptop; has regular access to a phone with text messaging capability; comfortable reading/viewing material on electronic devices; and able to attend in-person study visits and complete online questionnaires with their child over the full duration of the study.

### Recruitment

Several recruitment approaches (with bilingual materials) were utilized to enroll child-parent dyads. Recruitment letters were sent to the parent/guardian of eligible child patients (based on age, race/ethnicity, and BMI percentile criteria) who had received care at a community-based clinic (partnering organization) in upper Manhattan, NYC, within the last 2 years. We also intended to send recruitment letters to similar child patients of a government-insured medical clinic based in Upper Manhattan, NYC. However, barriers related to accessing patient data were encountered, thus preventing the use of this approach. Once the BMI percentile criterion was changed (see Study Population section above), recruitment approaches were expanded to include local community flyering in East Harlem/Upper Manhattan, posting inside/near local businesses, housing complexes, community centers, schools, and churches. Through several partnerships with local schools and community initiatives, recruitment efforts also occurred via tabling at community and school events.

Interested parents/guardians had the option to call, text, or email study staff to receive more information about the study. Those receiving recruitment letters also received a recruitment call to assess interest in the study and receive additional information. Interested parents/guardians completed a screening form via phone/email to determine study eligibility. Eligible participants were scheduled to attend a baseline (T1) visit where the child’s height and weight were measured to determine if their BMI percentile was at or above 5%.

To minimize attrition, child participants were compensated up to US $70 in gift cards and parent/guardian participants up to US $65 for completing data collection. Compensation was distributed in increasing amounts at each time point (T1: child US $10, parent/guardian US $15; T2: child US $15; T3: child US $20, parent/guardian US $20; and T4: child US $25, parent/guardian US $30). Each participant had the option to select a gift card from either a large department store retailer, a discount supermarket chain, a supermarket chain specializing in selling organic products, or a sporting goods retailer. Participants also received a round-trip metro card for any in-person study visits. If a dyad completed data collection at all 4 time points, they were entered into a raffle for a US $100 gift card.

### Sample Size

The sample size for this study was determined to reliably assess feasibility, acceptability, and preliminary efficacy of the intervention. We aimed to enroll a total sample size of 82 dyads (41 per group) [47,48], which allows for assessment of (1) intervention usage, usability, and feasibility/acceptability of study implementation and (2) both the within and between-group effect sizes as well as preliminary intervention efficacy based on mixed-models methodology (*d*=0.5, power=.80, alpha=.05, intraclass correlation coefficient=.6, 4 repeated measures), after taking into consideration estimated attrition of 20%. This sample size also allows to characterize potential sociodemographic moderators of the intervention as well as guide power calculations for a subsequent full-fledged RCT.

### Randomization

At baseline (after height and weight data were collected), eligible participants were randomized to either the experimental group or comparison group using a minimization allocation strategy (performed using the QMinim Web-based app created by Mahmoud Saghaei [49]). Randomization was performed at the dyad level and was balanced on child ethnicity (Hispanic or non-Hispanic) and BMI category (normal, overweight, or obese). Randomization was revealed at T4.

### Experimental Group Description

Interviews/focus groups, usability testing, and continuous quality improvement feedback on multiple prototypes with children and parents/guardians from our priority populations were used to inform development of *Intervention INC*, a theory-guided, interactive Web-based tool promoting healthy dietary behaviors (increased F/V and water intake), with the goal of reducing childhood obesity risk in black/African American and Latino preadolescents. Table 1 outlines the multiple phases and related research activities of the design and development process, which ultimately led to the final product that was tested in the pilot randomized trial. Details of the formative and development phases of *Intervention INC* are included in another manuscript (currently under review). *Intervention INC* comprises a 6-chapter comic with embedded goal-setting and messaging components. The tool is hosted on a password-protected website and optimized for use on tablet devices and touch-screen computer/laptop devices. All study participants received training on how to use the website at baseline.

#### Theoretical Framework

The narrative transportation theory (NTT), social cognitive theory (SCT), and health belief model (HBM) provided the theoretical framework for the *Intervention INC* tool. Comics, in particular manga comics (also known as Japanese comic art), are a unique form of multimodal narrative media that stimulate a reader’s attention by combining detailed visual images and text to create more of a subjective or personal viewpoint of a story [50]. The NTT explains how narrative communication, such as manga comics, could contribute to changes in health-related beliefs and behaviors by transporting the reader into the narrative world [51]. According to the NTT, transportation into a narrative world is believed to lead to acceptance of persuasive messages within a story through multiple mechanisms [52-54]. This theory also suggests that images are most impactful when they are embedded in a story rather than provided in isolation, as it could enhance the narrative influence [55]. Therefore, visual images relevant to the story’s message, such as those incorporated in manga comics, may further impact attitudes and beliefs. Furthermore, Latino and African American communities have strong storytelling traditions; thus, narrative-based health communication interventions could be effective with such populations, especially when culturally grounded messages and character experiences are depicted within relatable contexts [10].

SCT is a frequently used framework in effective dietary behavior change interventions [56,57], and it also lends explanation to ways in which a manga comic may influence health behavior in youth [7,8]. Exposure to characters in the storylines may facilitate observational learning and influence health behaviors, particularly when readers relate to the characters in the comics and consider them role models [58]. The development of entertainment-education narratives draws greatly on SCT by using role models to perform new behaviors [59-61]. SCT also supports self-regulatory behavior change procedures such as goal setting, self-monitoring, and feedback [58].

**Table 1:** Phases of *Intervention INC* tool design and development.

Phase	Activities	Objectives
Formative	Focus groups/interviews with children and parents	Identify factors influencing child dietary behaviors; assess technology use; and identify preferred comic storylines and characters
Development	Internal development of initial Web-based tool concepts; co-designing of Web-based tool content and design with children and parents; and usability testing of Web-based tool prototypes with children and parents	Draft initial tool outline and comic storyline/characters based on formative phase research; test and finalize acceptable and relatable tool components and comic content; and resolve tool usability issues identified during testing

**Figure 2:**
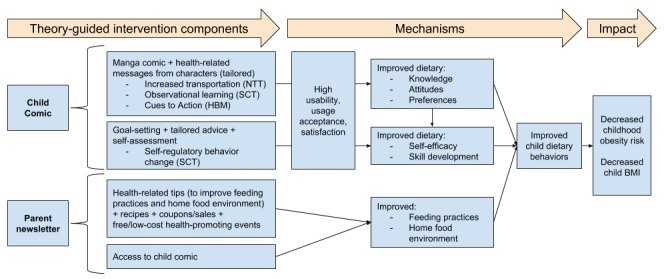
Conceptual framework of *Intervention INC*. BMI: body mass index; HBM: health belief model; NTT: narrative transportation theory; SCT: social cognitive theory.

The HBM construct of cues to action also guided tool development as it is a strategy to activate readiness for change and stimulate behavior change [56]. Thus, an innovative Web-based interactive tool that includes health messages delivered in a narrative comic format, tailored feedback, and cues to encourage behavior change, guided by the NTT, SCT, and HBM, might be an effective vehicle to promote healthy eating behaviors (see Figure 2).

#### Child Components

##### Comic

**Figure 3:**
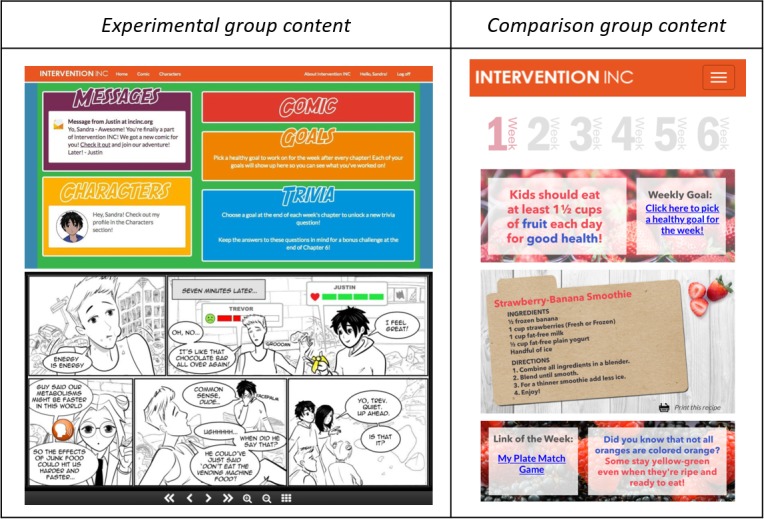
Experimental group (child) website homepage (top left) and snapshot of the comic (bottom left) and comparison group (child) Web-based newsletter example (right).

Children randomized to the experimental group were given access to a 6-chapter interactive nutrition comic titled “Game On” containing health messages focused on F/V and water consumption (see Figure 3). One chapter was made available at the beginning of each week for 6 weeks. Comic content was tailored (to include more information related to either F/V or water) based on responses to initial screening questions (asked at baseline) related to child F/V and water intake, child self-efficacy to increase F/V and water intake, and parent self-efficacy to support child in increasing F/V and water intake.

To ensure acceptability and relatability to our intended population, the comic storyline and its characters were informed by extensive formative research (highlighted in Table 1). Additional details related to how the formative research guided the design and development of the comic are described in another manuscript (currently under review). The comic depicts the story of a seemingly average high school student who discovers he has been tasked with the responsibility of saving the people of his world by battling an evil empire that has restricted access to F/V and water. With help from his friends, he must prepare for his epic battle by eating healthy foods and avoiding energy-dense, sugary foods/drinks. Personality traits, physical features of, and the communication/language used by characters in the comic were designed to be age-appropriate and culturally acceptable to both Latino and black urban children. Furthermore, scenarios in which the characters find themselves (eg, eating snacks in a bodega, racing to catch a subway for school) were intentionally incorporated into the story to model typical daily experiences of our intended population.

Health-related messaging was delivered through multiple mechanisms within the comic. Although the comic was primarily in black and white (the style typically observed in manga comics), health-promoting images related to F/V and water were featured in color for emphasis. In addition, characters modeled both healthy and unhealthy food behaviors, and the resulting benefits or consequences were depicted (in both image and text). Interactive features were also embedded into the comic (see Figure 3, bottom), such as orange-colored tap/click icons that either opened pop-up windows (highlighting health facts, food-related fun facts, or character information, which included favorite healthy foods/beverages) or prompted audio/visual effects to enhance engagement of the tool.

The comic was housed on a home page (see Figure 3, top), which comprised other sections including character profiles (highlighting demographic information for each character and personal fun facts) and trivia questions (combination of story-related and health-promoting questions, released as one per chapter). If all 6 trivia questions were answered correctly, the child received a prize of a downloadable screensaver image.

##### Goal Setting and Assessment

**Figure 4:**
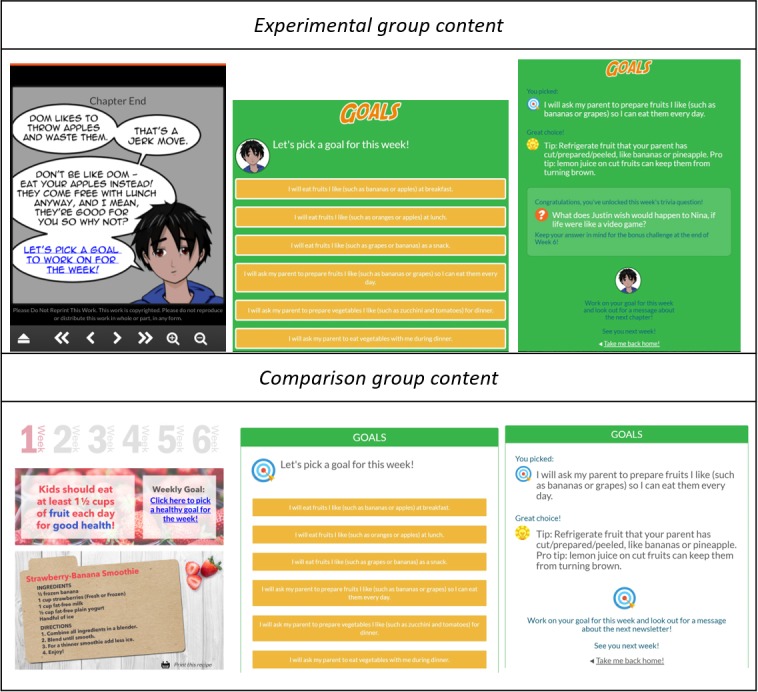
Experimental group (child) goal-setting component (top) with link in the character message at the end of each comic chapter (top left), list of goals (top center), and goal-specific tip (top right) and comparison group (child) goal-setting component (bottom) with link in the Web-based newsletter (bottom left), list of goals (bottom center), and goal-specific tip (bottom right).

At the end of each comic chapter, one of the comic characters was shown encouraging the child to select a goal to work on for the week (see Figure 4, top left). Clicking on the link in the message opened a new page where the child was able to choose from 8 tailored goals related to either encouraging increased F/V or water intake (see Figure 4, top center). Goals included ones that were child-focused (eg, “I will eat fruits I like [such as grapes or bananas] as a snack” or “I will drink a glass of water when I wake up each morning”) and ones that incorporated the parent (eg, “I will ask my parent to prepare vegetables I like [such as zucchini and tomatoes] for dinner” or “I will ask my parent to drink water with me during meals”). Once selected, a tailored tip was provided to assist the child in achieving that specific goal throughout the week (see Figure 4, top right).

Before selecting a new goal (at the end of the following week’s comic chapter), the child was prompted to self-assess how they did on the goal they chose and focused on for the past week by answering the following question: “How often did you do this in the last week?” (Never, Sometimes, Most of the Time, All the Time), and tailored feedback/encouragement was provided depending on the response chosen (ie, “Congrats! Keep up the good work!” or “Things take time - don’t give up!”). If the child selected “Most of the Time” or “All of the Time” in response to the goal assessment question, the child was rewarded with bonus comic content (eg, the backstory for a specific character). The child was then prompted to select another goal to work on for the following week. A total of 5 goals could be selected (no option to select goal at end of the last chapter) and worked on during the intervention period.

##### Text/Email Messages

**Figure 5:**
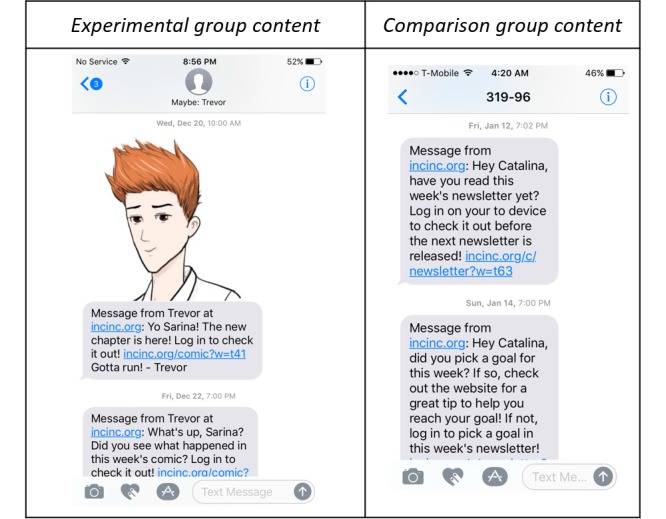
Examples of text messages sent to children in the experimental group (left) and comparison group (right).

A total of 4 messages were delivered to experimental group children each week (total of 23 messages throughout intervention). Messages included announcing the release of a new chapter by a comic character (see Figure 5, left), a reminder to read the comic, and a reminder to select a goal for each week. Messages were delivered via text and/or email based on participant preference identified during the baseline visit.

#### Parent/Guardian Components

##### Newsletters

**Figure 6:**
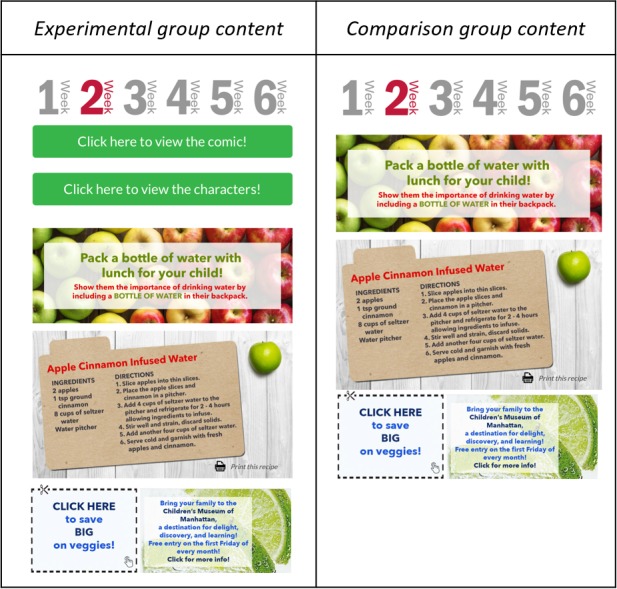
Examples of Web-based newsletters sent to parents in the experimental group (left) and comparison group (right).

Parents/guardians in the experimental group received 6 Web-based newsletters with similar health messages as their child (see Figure 6, left). Newsletter content comprised healthy recipes, healthy feedings tips for the family, links to coupons to support healthy eating, and links to fun community events (eg, fall and winter festivals). In addition, they were given access to the “Game On” comic and character profiles. Newsletters were translated into Spanish and provided to those parents who expressed a preference for Spanish-language materials.

##### Text/Email Messages

A total of 2 messages were delivered to experimental group parents/guardians (total of 11 messages throughout intervention) each week. Parents/guardians received messages announcing the release of a new newsletter. In addition, they received reminders to encourage their child to read the comic. Messages were delivered via text and/or email based on participant preference identified during baseline. Messages were translated into Spanish and provided to those parents who expressed a preference for Spanish-language materials.

### Comparison Group Description

The comparison group had access to Web-based tools similarly hosted on a password-protected website and optimized for use on tablet devices and touch-screen computer/laptop devices. All study participants received training on how to use the website at baseline.

#### Child Components

##### Newsletters

Children randomized to the comparison group received 6 Web-based newsletters with F/V or water information (see Figure 3, right). Similar to the comic, newsletter content was tailored (to include more information related to either F/V or water) based on responses to initial screening questions at baseline related to child F/V and water intake, child self-efficacy to increase F/V and water intake, and parent self-efficacy to support the child in increasing F/V and water intake. Newsletters were housed on a home page, and one was made available at the beginning of each week for 6 weeks. Health-related content for the comparison group was similar to that for the experimental group but was presented in a newsletter (didactic) format. The content comprised healthy eating tips, healthy recipes, diet-related knowledge/facts, health-promoting online games, and a link to selected weekly goals. Of note, comparison group participants were provided access to the “Game On” comic on completion of all data collection in April 2018.

##### Goal Setting and Assessment

Similar to children in the experimental group, children in the comparison group also had a goal-setting component (see Figure 4, bottom), wherein each week, the child could click the weekly goal link in the newsletter (see Figure 4, bottom left); select from 8 tailored goals to work on, related to either encouraging increased F/V or water intake (see Figure 4, bottom center); receive a tailored tip to provide assistance in achieving that specific goal throughout the week (see Figure 4, bottom right); and self-assess at the end of the week on how they did on the goal over the past week and receive feedback/encouragement. Unlike the goal-setting component in the experimental group, the format in which the goal setting was conducted in the comparison group was non-narrative.

##### Text/Email Messages

A total of 3 messages were delivered to comparison group children each week (total of 17 messages throughout intervention). Messages included announcing the release of a new newsletter, a reminder to read the newsletter, and a reminder to select a goal for each week (see Figure 5, right). Messages were delivered via text and/or email based on participant preference identified during the baseline visit.

#### Parent/Guardian Components

##### Newsletters

Parents/guardians of the comparison group received the same 6 Web-based newsletters (in English or Spanish) as that of the parents/guardians in the experimental group. However, parents/guardians in the comparison group were not given access to the “Game On” comic and character profiles (see Figure 6, right).

##### Text/Email Messages

One message (in English or Spanish) was delivered to comparison group parents/guardians (6 messages throughout intervention) each week announcing the release of a new newsletter. Messages were delivered via text and/or email based on participant preference identified during baseline.

### Measures

The measures collected are described in Table 2.

#### Feasibility/Acceptability Measures (Primary Measures)

##### Usage of Web-Based Tool

A custom-built platform was created to automatically log child and parent user details (created at baseline) and usage details (over 6 weeks of the intervention). User details included username, user type (parent or child), study group (experimental or comparison), type of tailored content (F/V or water), user language (English or Spanish), user email address, user mobile phone number, and start date/time (when username was created at baseline). Usage details included week number of intervention, link clicked, click time, platform used (eg, Mac OS, iPhone OS, Windows 7), and browser used and version (eg, Chrome 38.0, Safari 11.0). For child participants, additional usage data related to goal setting included weekly goal selected, date/time the goal was selected, evaluation at the end of the week (ie, “How often did you do this in the last week?”—Never, Sometimes, Most of the Time, All the Time), and date/time the goal was evaluated.

##### Usability of Web-Based Tool

Questionnaires were administered to the child and parent at several time points throughout the study. Usability was assessed with an adapted version of the System Usability Scale (SUS) [62]; Usefulness, Satisfaction, and Ease-of-use questionnaire (USE) [63]; and a 26-item acceptability/usability measure by Ben-Zeev et al [64] to assess 5 usability domains: usability, usefulness, ease of use, ease of learning, and satisfaction. The child’s questionnaire comprised 30 usability questions—10 questions from SUS, 15 from USE, and 5 from the acceptability/usability measure. On the basis of pilot testing with Latino and black children, modifications were made to tailor the questionnaire according to the literacy levels of our intended population. For example, the item “I found the system very cumbersome to use” was replaced with “I found it awkward to use” and “I would imagine most people would learn to use this system very quickly” was changed to “I think most people my age would learn to use it very quickly.” The parent questionnaire comprised 9 usability questions—2 questions from SUS, 3 questions from USE, and 4 questions from the acceptability/usability measure. On the basis of pilot testing, 1 item was modified. Similar to the child questionnaire, the item “I found the system very cumbersome to use” was replaced with “I found it awkward to use.” As the content of the parent component was presented in a more didactic format compared with that of the child component, fewer usability questions were relevant to include in the parent questionnaire. In addition, usability data in the form of qualitative interviews with child participants (at T2, T3, and T4) and parent participants (at T3 and T4) were collected to supplement quantitative usage and usability questionnaire data.

**Table 2:** Key measures, data source, and time of assessment. All questionnaire items were either taken from or directly informed by validated questionnaires.

Measures		Data source	Time points				
			T1^a^	T2^b^	T3^c^	T4^d^	O^e^
**Feasibility/acceptability measures (primary measures)**							
	Usage of Web-based tool	Tracking system (internally created)	—^f^	—	—	—	C^g^/P^h^
	Usability of Web-based tool	Interview, questionnaire items [62-64]	—	C	C/P	C/P	—
	Feasibility of study implementation	Process data (eg, recruitment, attrition) [65,66]	—	—	—	C/P	S^i^
**Outcome measures (secondary measures)**							
	Dietary knowledge and attitudes	Questionnaire items [67,68]	C	C	C	C	—
	Dietary intake	Questionnaire items [69,70]	C	C	C	C	—
	Anthropometric measures	Digital stadiometer, body composition monitor	C	—	—	C	—
	Feeding practices	Questionnaire items [71]	P	—	P	P	—
	Home food environment	Questionnaire items [72]	P	—	P	P	—

^a^T1: Time points indicated by baseline.

^b^T2: Time points indicated by midpoint (3 weeks postbaseline).

^c^T3: Time points indicated by endpoint (6 weeks postbaseline).

^d^T4: Time points indicated by follow-up (3 months postintervention).

^e^O: Ongoing throughout intervention period.

^f^Indicates measures that were not collected at specific timepoints.

^g^C: Data collected from child.

^h^P: Data collected from parent/guardian.

^i^S: Data collected from study staff (internal).

##### Feasibility of Study Implementation

Process data collected throughout the study assessed the feasibility of implementing the study [65,66]. These data included quantitative/qualitative measures of recruitment and retention (ie, enrollment rate, restrictiveness of eligibility criteria, attrition rate), assessment of resource capacity (ie, staff hours needed for recruitment, participant communication approaches), data collection (ie, length of time to complete online questionnaires), and data reliability (ie, study staff adherence to protocol). Satisfaction with study participation was assessed through questionnaire items with child and parent participants at T4 (ie, frequency and format of communication and study visit scheduling with study staff) as well as qualitative observations of child participants at T1 and T4 while completing questionnaires (ie, verbal and nonverbal expressions indicating frustration, boredom, and confusion).

#### Outcome Measures (Secondary Measures)

##### Child Dietary Knowledge and Attitudes

Child participants completed a questionnaire related to knowledge, outcome expectations, self-efficacy, behavioral intention, attitudes and preferences regarding behaviors associated with F/V, water, junk food, and sugary drinks. A total of 6 questions addressed knowledge (ie, I should eat 1 cup of fruit each day for good health), 20 questions addressed outcome expectancies (ie, Eating vegetables every day will keep me from getting sick), 9 questions addressed self-efficacy (ie, If I decide to not eat junk food every day, I can do it), 10 questions addressed intention (ie, If my parent offers me water, I will drink it), 15 questions addressed attitudes (ie, I think sugary drinks are cool), and 24 questions addressed preferences (ie, Which of the following fruits do you like or dislike?). A total of 84 questions were informed by and modified from the validated ProChildren questionnaire [67] and the validated Reynolds questionnaire [68]. Modifications to wording were made to ensure questions were appropriate for this study and to adjust for literacy levels of our intended population. A recent study conducted among Latino children also adapted the ProChildren questionnaire to measure children’s self-efficacy for eating fruits and vegetables and for consuming water [73].

##### Child Dietary Intake

Child participants completed a questionnaire that assessed the frequency of consumption of F/V, water, junk food, and sugary drinks during the past 7 days. This 17-item questionnaire comprised 8 questions (6 directly from and 2 informed by) from the validated 2017 Youth Risk Behavior Surveillance System (YRBSS) questionnaire [69], which was created to monitor obesity prevalence and related behaviors, among other priority adolescent health issues. Nationally representative samples of students along with selected large urban school districts are engaged in the data collection process. Other studies conducted specifically with black and Latino children exploring dietary and physical activity behaviors have also utilized items from the YRBSS questionnaire [74,75]. Moreover, 6 items were informed by the validated Beverage and Snack Questionnaire, which was tested with a diverse group of children as approximately 45% were from minority populations [70], and it was used in a more recent study that evaluated the efficacy of a serious game on low-income urban public school children’s dietary behaviors [76]. A total of 3 items were internally created (related to assessing the intake of different types of water).

##### Child Anthropometric Measures

Height and weight of child participants were measured using standardized methods [77]. Height was measured to the nearest 1/8 inch using a digital stadiometer (SECA 264), with the participant fully erect, without shoes, feet together, head in the Frankfort plane, and at the end of a deep inhalation. Weight and body composition were measured using a body composition monitor (Tanita MC-780U) wearing lightweight clothes and without socks and shoes. Weight was measured to the nearest 0.2 lb. Height and weight were measured in duplicate and recorded. A third measurement was taken if there was any uncertainty on the accuracy of height or weight measurements (eg, if height measurements differed more than 0.5 inch). An average of the measurements was used for the BMI calculations. The Centers for Disease Control and Prevention BMI percentile calculator was used to determine BMI percentage [78]. If needed, the average height was rounded down to the nearest eighth of an inch, and the average weight was rounded down to the nearest quarter pound, to accommodate the calculator’s units of measurement.

##### Parent Feeding Practices

Parent participants completed a questionnaire that asked about multiple parental feeding practices, specifically including 6 questions related to environment (ie, I offer a second helping of vegetables to my child during meals at home), 4 related to involvement in purchasing/preparing food (ie, I allow my child to help prepare fruit and vegetable dishes for family meals), 7 related to encouragement (ie, I encourage my child to drink water drinks [unsweetened] before sugary beverages), 8 related to modeling (ie, I model drinking water for my child even if it is not my favorite), and 2 related to teaching about healthy food practices (ie, I discuss with my child why it’s important to eat fruits and vegetables.). The 27-item questionnaire was informed by the validated Comprehensive Feeding Practices Questionnaire [71], which has been used in previous studies with low-income Hispanic parents and African American fathers [79,80].

##### Home Food Environment

Parent participants completed a questionnaire related to the availability of fruits, vegetables, and water in their home and how often they store fruits, vegetables, and water in a place easily seen by their child. The questionnaire comprised 6 questions and was informed by the validated Home Environment survey [72].

#### Potential Confounders

Demographic factors such as age, gender, race/ethnicity, and whether the United States was the country of birth for both the child and parent were collected. Additional child measures included grade, technology use, physical activity, sedentary behavior, and perceived health. Parental measures also included marital status, education level, household income, household profile, Supplemental Nutrition Assistance Program participation, child participation in school breakfast and lunch program, and perceived health. These measures were collected at T1.

### Data Management

A manual of procedures, including protocols related to data collection and storage, was developed at the outset of the study and refined continuously with input from all study staff. Study staff involved in collecting data were trained in implementing all procedures. Data collection and management procedures were reviewed at study staff meetings throughout the intervention period to ensure that they were followed with fidelity and to also address any issues or barriers to implementation.

Data collected in this study includes both quantitative data (auto-generated website usage data, online questionnaires, and anthropometric measures) and qualitative data (interviews). To ensure generated data are reliable, valid, and usable, the study staff used validated questionnaire items (or questionnaire items informed by validated questionnaires) and best practices for questionnaire, interview, and anthropometric data collection. Quantitative data were downloaded in spreadsheet format at least twice weekly, and qualitative data were downloaded as audio files weekly. All data were uploaded to password-protected institutional servers. Data were checked regularly to ensure the accuracy of data capture. A data dictionary that includes original items, answer choices, scoring/coding of answers, scoring of scales, and examples was created to ensure that all project data are accurately and readily usable and to aid in data analysis.

### Data Analysis

#### Quantitative Data

Usage and usability of the Web-based tool, along with the feasibility of study implementation, will be assessed using descriptive analyses. As usage data are key to understanding technology-based behavioral intervention dose and also how participants engage with the Web-based tool itself [81], patterns of usage by children and parents/guardians will be described/calculated in multiple ways including as a binary variable by week (eg, did child/parent visit the website in week 1), as an ordered variable based on usage over 6 weeks (eg, high use, low use, and minimal use), or as a continuous variable measured as number of times visited over 6 weeks. Previous studies have similarly assessed patterns of usage when analyzing user engagement with a website or app in an intervention [82-85]. Both usage and usability data will also be used to stratify analysis to determine if there are any significant associations between individual demographic characteristics and health-related outcomes. These data will have high relevance for interpretation of outcome data and further inform refinement and enhancement of the intervention.

The changes in study outcomes within and between groups will be examined using mixed-models methodology with repeated assessments (T1, T2, T3, and T4), condition (experimental / comparison), and time by condition interaction. Both within- and between-group effect sizes will be calculated for all study measures to assess the magnitude of intervention effects overall and by potential moderators and inform subsequent larger RCTs. To control for multiple comparisons, *P* values will be evaluated based on the false discovery rate [86]. All analyses will be conducted using an intent-to-treat approach.

#### Qualitative Data

Audio files of interviews conducted with children and parents/guardians at T2, T3, and T4 will be transcribed. Inductive and deductive processes will be used to analyze qualitative data collected from interviews with child participants (at T2, T3, and T4) and parent participants (at T3 and T4) as well as qualitative observations (detailed notes) during in-person study sessions at T1 and T4. Using a content analysis approach [87], transcribed audio files and field notes will be coded by at least 2 independent reviewers and reviewed to identify trends and recurring themes, especially related to barriers and facilitators to use and adoption of the *Intervention INC* Web-based tool. A qualitative analysis software will be used to assist with organizing, coding, and analyzing transcripts and notes.

## Results

A total of 89 child-parent dyads were enrolled into the study on a rolling basis between August and November 2017. The dyads were randomized to either the experimental (n=44) or comparison (n=45) group. The pilot RCT was concluded as of April 2018, and data analysis is currently underway.

## Discussion

### Implications and Strengths

The *Intervention INC* Web-based interactive tool was developed to help engage low-income, minority children to change individual dietary behaviors and provide parents information and resources to improve feeding practices and promote a supportive home food environment, with the ultimate goal of reducing childhood obesity risk.

To the best of our knowledge, this is one of the first studies to explore the potential impact of an interactive Web-based tool specifically designed by and for at-risk, minority preadolescents. There is a distinct lack of effective health promotion tools that have been culturally tailored to meet the needs and preferences of populations with disproportionate rates of chronic disease [88], even fewer have been developed for children [18]. Our tool aims to address this as it has been specifically designed for black/African American and Latino children, who are at the greatest risk of childhood obesity. Furthermore, our intervention engages parents who play a significant role in shaping the home food environment and influencing child dietary behaviors [89,90]. It is well documented that sustained engagement of both children and parents in long-term interventions is a major challenge, particularly within at-risk populations [91,92].

*Intervention INC* is unique, given its innovative narrative and interactive Web-based approach to enhance adoption of the tool with hard-to-reach, at-risk populations. Embedded points of interactivity such as the pop-up features and special effects might further enhance engagement of the tool, thus potentially increasing the exposure of the intervention through additional opportunities to deliver meaningful health information. Assignment to a tailored track was based on initial screening at baseline to assess child F/V and water intake and child/parent self-efficacy related to F/V and water intake. Results of the screening process identified which at-risk behavior (F/V or water intake) was targeted during the intervention.

Notably, user-centered approaches were used throughout formative and development stages of this intervention, including co-development of the storyline and other content with children and parents and the use of multiple usability sessions to inform refinement of the tool. Although several studies have emphasized how usability testing can improve technology-enhanced tools [93-95], few have conducted usability testing methods for health promotion tools with youth users [96-98]. Usability testing is a crucial step in the development of online health tools to ensure that they are accessible, understandable, and useful to end users and are delivered in an efficient, effective, satisfying, and culturally competent manner [99].

Our study has numerous strengths, which include objective measures such as tool usage (process) and BMI (outcome). Importantly, the collection and analysis of detailed usage data allow for the potential to identify mechanisms of change, that is, which components of the *Intervention INC* tool might be contributing to any observed dietary-related changes. Our study has also incorporated multiple strategies to minimize attrition, which include partnering with community organizations, basing the study site within our priority community, recruiting bilingual research team members who come from similar communities as our participants, providing incentives comprising gift cards to local stores (dispersed in increasing amounts) throughout participation period, and sending emails with recipes during the 3-month follow-up period.

### Limitations

This pilot study has limitations, which need to be considered. Initial recruitment challenges led to modifications in BMI eligibility criteria. In addition to overweight/obese children, healthy-weight children were also recruited. Not only could changes in BMI criteria dilute any anthropometric changes, but such recruitment challenges will also have implications for a larger scale randomized trial. Participants were recruited on a rolling basis over the course of 3 months; therefore, seasonality might bias various health-related behaviors, and thus our findings. The comparison group received similar health information (in non-narrative form), which may result in differences not being observed between groups. In addition, the comparison group (both child and parent) received 1 less text/email weekly reminder, which may influence tool usage, though this is likely to be minimal. Self-reported data from both the children and parents have been collected, which pose a validity risk, due to intentional/unintentional misreporting. However, our study design allows for children and parents to complete surveys at home as opposed to the study site with staff, which may contribute to decreased social desirability bias.

### Conclusions

This study aims to implement an innovative approach to deliver healthy dietary messages and resources to at-risk minority children and their parents. If found to be acceptable, engaging, and feasible, a larger RCT with the *Intervention INC* Web-based comic tool will be conducted to assess its efficacy related to improving child dietary behaviors, child health outcomes, parent feeding practices, and the home food environment.

## Appendix

Multimedia Appendix 1 Peer-reviewer report from the Agency for Healthcare Research and Quality.

## Abbreviations

AHRQ: Agency for Healthcare Research and Quality
BMI: body mass index
F/V: fruit/vegetable
HBM: health belief model
INC: Interactive Nutrition Comics for urban, minority preadolescents
mHealth: mobile health
NTT: narrative transportation theory
NYC: New York City
RCT: randomized controlled trial
SCT: social cognitive theory
SUS: System Usability Scale
USE: Usefulness, Satisfaction, and Ease-of-use questionnaire
YRBSS: Youth Risk Behavioral Surveillance
