# Supplementary material for: Testing a Web-Based Interactive Comic Tool to Decrease Obesity Risk Among Minority Preadolescents: Protocol for a Pilot Randomized Control Trial
Source: JMIR Res Protoc. 2018 Nov 9;7(11):e10682. doi: 10.2196/10682 (PMC6251980; doi:10.2196/10682)
Supplement: Multimedia Appendix 1 [file resprot_v7i11e10682_app1.pdf]

SUMMARY STATEMENT
(Privileged Communication)

PROGRAM CONTACT:
Amy Lindinha
(301) 427-1614
amy.lindinha@ahrq.hhs.gov

Release Date: **11/10/2015**

Application Number: **1 R21 HS024117-01A1**

Principal Investigator

LEUNG, MAY MAY PHD

Applicant Organization: HUNTER COLLEGE

Review Group: **HITR**
Healthcare Information Technology Research

Meeting Date: **10/22/2015**
Council: **JAN 2016**
Requested Start: **01/01/2016**

RFA/PA: **PA14-001**
PCC: **CEPI**

Project Title: **Intervention INC: Interactive Nutrition Comics for urban minority youth**

SRG Action: **Impact Score: 29 Percentile: 8**

Human Subjects: **30-Human subjects involved - Certified, no SRG concerns**
Animal Subjects: **10-No live vertebrate animals involved for competing appl.**
Gender: **1A-Both genders, scientifically acceptable**
Minority: **1A-Minorities and non-minorities, scientifically acceptable**
Clinical Research - not NIH-defined Phase III Trial

Project Year	Direct Costs Requested	Estimated Total Cost
1	87,060	141,442
2	97,594	158,555
TOTAL	184,654	299,997

ADMINISTRATIVE BUDGET NOTE: The budget shown is the requested budget and has not been adjusted to reflect any recommendations made by reviewers. If an award is planned, the costs will be calculated by Agency grants management staff based on the recommendations outlined below in the COMMITTEE BUDGET RECOMMENDATIONS section.

RESUME AND SUMMARY OF DISCUSSION: This R21 health services research grant resubmission from Dr. May May Leung, from Hunter College, in NYC, proposes a pilot and feasibility study for urban minority youth, which aims to design and develop a human-centered, interactive, internet/mobile-enabled dietary self-management intervention focused on reducing childhood obesity risk. The reviewers agreed that the proposed study is significant since obesity is a growing problem among children today and leads to illness, development of diabetes and has long lasting impacts on individuals, both in physical and psychosocial ways. While electronic interventions focused on childhood obesity prevention have been developed, sustainable results have been limited. The reviewers added that the applicant plans to address knowledge gaps in the usability and usefulness of HIT in changing behavior, including its potential for adoption, engagement, and sustained use, as well as its effectiveness in reducing childhood obesity. The study seeks to engage children and parents in the customization of a program, offers a pilot program and feasibility study of the intended device. The application includes a well-organized theoretical framework including the Human Centered approach, narrative-based approach using narrative transportation theory and social cognitive theory, to develop the Interactive Nutrition Comics (INC) intervention. The applicant has addressed many of the concerns reviewers highlighted in the previous application that improve the study impact. However, the reviewers also noted that while the parents have a major role in what food is available for the child, their intervention is minimal, which may negate the potential impact of the study. Also, the intervention development, implementation, follow-up with multiple surveys seems very complex for the study population of children ages 10-12, and it is time consuming. There we also some concerns about feasibility of the project within 2 years. Overall, the reviewers recommended this application for further consideration with an outstanding to excellent level of enthusiasm.

DESCRIPTION (provided by applicant): Childhood obesity continues to be a serious clinical and public health issue in the United States, with disproportionate rates among low-income, minority children. This complex epidemic has been attributed to various dietary behaviors, such as decreased fruit and vegetable consumption and increased sugary drink and energy-dense snack consumption. As food preferences established in childhood often continue into adulthood, it is important to intervene at this critical period. Effective, yet innovative interventions need to be designed to capture the attention of children living in a multi-media environment. Interactive, technology-based approaches are emerging as promising tools for supporting health-behavior change. However, knowledge gaps in the usability and usefulness of such applications still exist, limiting their potential effectiveness. To fill this gap, we propose to conduct a pilot and feasibility study entitled Intervention INC: Interactive Nutrition Comics for urban minority youth, which aims to design and develop a human-centered, interactive, internet/mobile-enabled dietary self-management intervention focused on reducing childhood obesity risk. Using an innovative narrative-based comic and a human-centered approach during the design and development process, this intervention will be tailored to meet the needs and preferences of the at-risk population of low-income, minority, inner-city children ages 10 to 12. In Aims 1 and 2, formative research will be conducted with the intended population and their parents to inform the development of an engaging and appealing intervention, while Aim 3 will assess the intervention's feasibility and acceptability and explore if this interactive, internet/mobile-enabled dietary self-management intervention improves knowledge, attitudes and food preferences associated with the targeted behaviors to reduce childhood obesity risk, using a 2-group randomized study design. We hypothesize that children who participate in an interactive, internet/mobile-enabled dietary self-management intervention will report improved knowledge, outcome expectancies, self-efficacy and food preferences related to targeted dietary behaviors than children in a control group, who will only receive internet/mobile internet access. We believe that Intervention INC is unique given its focus on innovative approaches to enhance adoption and engagement. It also has strong

PUBLIC HEALTH RELEVANCE: Intervention INC: Interactive Nutrition Comics for urban minority youth is a pilot and feasibility study of an innovative human-centered, interactive, internet/mobile-enabled dietary self-management intervention, tailored to the needs and preferences of low-income,

minority, inner-city children ages 10 to 12, a high risk population for childhood obesity. The proposed study will examine usability and usefulness of the newly designed intervention, with the aim of enhancing its adoption, engagement and use, and explore the intervention's impact on knowledge, attitudes and food preferences related to childhood obesity risk. Intervention INC has strong public health relevance as it aims to not only reduce childhood obesity risk, but has significant long-term implications for improved overall quality of life and reduction of adult morbidity and mortality risk for the vulnerable urban minority population.

CRITIQUE NOTE: The sections that follow are the essentially unedited, verbatim comments of the individual committee members assigned to review this application. The attached commentaries may not necessarily reflect the position of the reviewers at the close of group discussion, nor the final majority opinion of the group. The above RESUME/SUMMARY OF DISCUSSION represents the evaluation of the application by the entire committee.

CRITIQUE 1

Significance:	2
Investigator(s):	2
Innovation:	2
Approach:	4
Environment:	1

Overall Impact:
Strengths
- Childhood obesity is an important public health problem in the US especially among low-income, minority populations and is associated with significant short and long-term economic, physical, and psychosocial consequences, as well as increased child's risk of adult morbidity and mortality.
- While electronic interventions focused on childhood obesity prevention have been developed, sustainable results are limited. The applicant plans to address knowledge gaps in the usability and usefulness of HIT in changing behavior, including its potential for adoption, engagement, and sustained use, as well as its effectiveness in reducing childhood obesity.
- The applicant has addressed many of the concerns reviewers highlighted in the previous application that improve the study impact.
- The application includes a well-organized theoretical framework including the Human Centered (HC) approach, narrative-based approach using narrative transportation theory (NTT), and social cognitive theory (SCT), to develop the INC intervention.

Weaknesses
- One element of the INC intervention relies on the parents to send encouragement message to their children. This assumes underserved parent participants have optimal knowledge and self-efficacy about obesity risk management to help their children achieve optimal behavior change. Often parents are the problem of the child obesity despite education provided without any active intervention that also involves them. The intervention on the parent is underdeveloped.
- The intervention development, implementation, follow-up with multiple surveys seems very complex for the study population (children 10-12) and time consuming and there are concerns about feasibility of the project within 2 years.

1. Significance:
Strengths
- Childhood obesity is an important public health problem in the US especially among low-income, minority populations and is associated with significant short and long-term economic,

physical, and psychosocial consequences, as well as increased child's risk of adult morbidity and mortality.
- The intervention to be designed will use the opportunity presented by children who are living in a multi-media environment (e.g. video games, computers and television) to capture their attention.
- If successful, the proposal can lead to a new approach of technology-based health promotion interventions to engage children in self-management of obesity.
- The investigators have experience with the field of study and provided strong preliminary data, which demonstrates their ability to engage children in such study and to successfully carry out the project.

Weaknesses
- The intervention development, implementation, follow-up with multiple surveys seems complex for the study population (children 10-12) and time consuming, and there are concerns about feasibility of completing this project within 2 years.

2. Investigators:
Strengths
- The applicant (Dr. Leung) is Assistant Professor, Nutrition Program, City University of New York School of Public Health and Hunter College. She is an early stage investigator and applied nutrition interventionist, who has experience developing and evaluating innovative health communication and community-based interventions, to reduce childhood obesity risk in vulnerable, minority youth populations. She also has experience using community-based participatory research methods and tailoring of interventions to the needs and preferences of the intended population. The applicant is supported by Dr. Joshi, an experience researcher in Biomedical informatics.
- Dr. Allison Gorman is an Assistant Professor of Pediatrics, will act as a consultant for the project and the primary contact of the project at Weill Cornell Medical College (WCMC). She will provide consultation related to the clinical perspective in development and implementation of the intervention, as well as logistical support and assistance in subject recruitment efforts at WCMC.

Weaknesses
- No weaknesses noted.

3. Innovation:
Strengths
- Use of narrative-based approach with comics related to lifestyle choices to increase persuasion to low income minority youth of the story's health messages using an online application seems innovative.
- Use of both NTT and SCT in the development of the INC intervention to enhance acceptability and use of the program.

Weaknesses
- Comic like approach with avatar has been used in obesity, although previous studies have not included NTT.

4. Approach:
Strengths
- The project will use well organized interdisciplinary theoretical framework including the Human Centered (HC) approach, narrative-based approach using narrative transportation theory (NTT), and social cognitive theory (SCT) to involve potential users, develop the INC intervention and increase the likelihood of the application use and acceptance by children.
- Use of a randomized control trial to test the effectiveness of the application is strong.

Weaknesses

- One element of the INC intervention relies on the parents to send encouragement message to their children. This assumes underserved parent participants have optimal knowledge and self-efficacy about obesity risk management to help their children achieve optimal behavior change. Often parents are the problem of the child obesity despite education provided without any active intervention that also involves them. The intervention on the parent is underdeveloped.
- The intervention development, implementation, follow-up with multiple surveys seems very complex for the study population (children 10-12) and time consuming and there are concerns about feasibility of the project within 2 years.

5. Environment:
Strengths

- The research environment of Hunter College of the City University of New York (CUNY) and The CUNY School of Public Health as well as of The Children's Aid Society (which operates a network of community centers, community schools, teen centers, early childhood centers, and medical, dental and mental health clinics in geographic pockets of poverty) throughout New York City, is excellent. The applicant has letters from involved organizations supporting the project.
- Collaboration with Weill Cornell Medical College is strong.

Weaknesses

- No weaknesses noted.

Privacy and Security Protections in the Development and Implementation of Health IT System*:*
Addressed.

Protection of Human Subjects from Research Risks: Acceptable.

Inclusion of Women and Minority Subjects: Acceptable.
Strengths

- This study targets low-income (both males and females) minority children 10-12 years of age.

Weaknesses

- No weaknesses noted.

Inclusion of AHRQ Priority Populations: Acceptable.

Degree of Responsiveness: The application is responsive.

Budget and Period of Support: Acceptable.

Resubmission Applications:
Strengths

- The applicant has addressed many of the concerns reviewers highlighted in the previous application, including: accessibility of computers and internets in low-income populations, accessibility and availability of healthy food choice for the study population, rationale for targeting children 10-12 years old, difference between their intervention comics and avatars (which are not narrative), description on how they will improve the existing print form to computer-based intervention, methods to engage children in the focus group, addition of parental education about the intervention, previous success recruiting children in a study, specifying that study population criteria including use of BMI, assessment of child and parent literacy, assessment of barriers and facilitators of the intervention use, sample size calculation which consider attrition etc.

Weaknesses

- No weaknesses noted.

CRITIQUE 2

Significance: 2
Investigator(s): 2
Innovation: 3
Approach: 3
Environment: 2

Overall Impact:
Strengths
- Childhood obesity in low income children is important to address.
- The investigative team is well-qualified.
- The approach is strong.
- The proposal addressed most of the prior concerns.

Weaknesses
- The parental intervention is minimal and parents will have a major role in what food is available for the child.

1. Significance:
Strengths
- The focus of this application, developing a program to combat obesity in low income children, is an important focus.

Weaknesses
- No weaknesses noted.

2. Investigators:
Strengths
- The investigators have relevant expertise and experience. They have successfully used the content and the delivery methods previously and are combining them for this project.

Weaknesses
- No weaknesses noted.

3. Innovation:
Strengths
- An interactive comic book style intervention is potentially innovative.

Weaknesses
- The methods have been used by the investigators previously, so neither the comic book nor the computer-based intervention is really novel. The only novelty is in their combination.

4. Approach:
Strengths
- The authors have designed a randomized trial and have a good plan for usability testing and user-centered design.

Weaknesses
- The parental intervention is minimal and parents will have a major role in what food is available for the child. This may negate the potential impact of the study.

5. Environment:
Strengths

- The environment is very appropriate for this intervention, but it is a difficult population to get sustained engagement.

Weaknesses

- No weaknesses noted.

Privacy and Security Protections in the Development and Implementation of Health IT System*:* The privacy and security of the research database is described and is adequate, but there is no security plan described for the web-based application the patients will be using.

Protection of Human Subjects from Research Risks: Acceptable.

Inclusion of Women and Minority Subjects: Acceptable.

Inclusion of AHRQ Priority Populations: Acceptable.

Degree of Responsiveness: This application is moderately responsive.

Budget and Period of Support: Budget is adequate.

Resubmission Applications: The applicant responded well to the previous comments.

CRITIQUE 3

Significance:	2
Investigator(s):	2
Innovation:	3
Approach:	4
Environment:	1

Overall Impact:
Strengths

- This study addresses an important issue in helping minority groups be more knowledgeable about nutrition and making efforts to engage children and parents in that effort. Obesity is a national problem that is getting worsening.

Weaknesses

- The study may be better completed in parts.
- There are methodological concerns that exist (see approach section).

1. Significance:
Strengths

- Obesity is a growing problem among children today and leads to illness, development of diabetes and has long lasting impacts on individuals, both in physical and psychosocial ways.
- This application identifies a gap (opportunity) for children to learn to self-manage nutrition in healthy ways by means of HIT programs on mobile devices or telephones.
- This study seeks to engage children and parents in the customization of a program, offers a pilot program and feasibility study of the intended device.

Weaknesses

- The study in its proposed format seems to be rather complex.
- The intention of customized comics is not articulated well. Will there be a variety of comics developed? What about copyright infringement for published comics?
- Methodology needs further clarification.

- The comic book approach will likely inspire child engagement.

2. Investigators:
Strengths
- New PD/PI however has experience with the management of multiple funding opportunities.
- The PD/PI is appropriately trained and has a wealth of knowledge and experience about nutrition and children.

Weaknesses
- An investigator that specialized in health literacy would be an important contributor to strengthen this study.

3. Innovation:
Strengths
- This is a new type of tool based on a narrative approach to improve nutrition in the underserved minority population.

Weaknesses
- There are many electronic tools for children to use to assist with teaching and learning of nutrition.
- It seems that children of this age like to solve problems using technology. How is this incorporated into the design (if at all)?

4. Approach:
Strengths
- Randomized control trial is a stronger study.
- Seeks to use multiple approaches to engage children and their parents in preventing/reducing childhood obesity.

Weaknesses
- The overall plan may be difficult to accomplish in the time frame indicated.
- For the control group, they will have Internet/Mobile Internet access to what? Who is providing the mobile Internet or Internet access?
- Is there any chance that the focus groups could actually make the parents feel like they are not the best parents because of what they feed their children?
- It is unlikely that parents of underserved minorities and their child will be able to understand work-flow and design principles at this point.
- It appears that this device is circumventing face to face communication between parents and their children. Is that what is intended?
- The Appendix D: Aim 1 Sample focus Group Questions as wording that will be difficult for parents and children to read and understand. Consider checking with someone that is keen on health literacy. (e.g. affecting/influencing, food behaviors, health risks)
- Behavior can be thought in terms of negative consequences. Perhaps food choices are easier than food behavior.
- Some of the programing exists in other electronic programs (Fitbit and others).
- How will expected changes in body composition be accounted for in the study?

5. Environment:
Strengths
- The environment is appropriate for this study.

Weaknesses
- No weaknesses noted.

Privacy and Security Protections in the Development and Implementation of Health IT System*:*

Data and safety monitoring plan –none. Since the study calls for getting the data and analyzing it, the data must be de-identified and some measure to ensure that the data is maintained in a secure environment.

Protection of Human Subjects from Research Risks: Acceptable.

Inclusion of Women and Minority Subjects:
Strengths
- This study includes women as parents of the children. The overall population for this study is minorities' children ages 10-12. Male/female ratio is equal.

Weaknesses
- No weaknesses noted.

Inclusion of AHRQ Priority Populations:
Strengths
- Female and male parents as participants. Children who are African American and Hispanic.

Weaknesses
- No weaknesses noted.

Degree of Responsiveness:
Strengths
- The application is responsive to the FOA. The specific area is the usefulness of the Health IT system or application.

Weaknesses
- No weaknesses noted.

Budget and Period of Support: Budget is appropriate. Perhaps there is one error on page 73

THE FOLLOWING RESUME SECTIONS WERE PREPARED BY THE SCIENTIFIC REVIEW ADMINISTRATOR TO SUMMARIZE THE OUTCOME OF DISCUSSIONS OF THE REVIEW COMMITTEE ON THE FOLLOWING ISSUES:

PROTECTION OF HUMAN SUBJECTS (Resume): ACCEPTABLE.

INCLUSION OF WOMEN PLAN (Resume): ACCEPTABLE

INCLUSION OF MINORITIES PLAN (Resume): ACCEPTABLE

INCLUSION OF AHRQ PRIORITY POPULATIONS PLAN (Resume): ACCEPTABLE

COMMITTEE BUDGET RECOMMENDATIONS: The budget was recommended as requested
